# Identification of prognostic stemness biomarkers in colon adenocarcinoma drug resistance

**DOI:** 10.1186/s12863-022-01063-9

**Published:** 2022-07-06

**Authors:** Ziyue Li, Jierong Chen, Dandan Zhu, Xiaoxiao Wang, Jace Chen, Yu Zhang, Qizhou Lian, Bing Gu

**Affiliations:** 1 Cord Blood Bank, Guangzhou Institute of Eugenics and Perinatology, Guangzhou Women and Children’s Medical Center, Guangzhou Medical University, Guangzhou, 510000 China; 2 Division of Laboratory Medicine, Guangdong Provincial People’s Hospital, Guangdong Academy of Medical Sciences, 106 Zhongshan 2nd Rd, Yuexiu District, Guangzhou, Guangdong 510000 P. R. China; 3 Laboratory Schools, the University of Chicago, Chicago, IL USA

**Keywords:** Colon adenocarcinoma, Cancer stem cell, Chemoresistance, Prognosis, Biomarkers

## Abstract

**Background:**

Colon adenocarcinoma (COAD) is one of the leading causes of death worldwide. Cancer stem cells (CSCs) are vital for COAD chemoresistance and recurrence, however little is known about stem cell-related biomarkers in drug resistance and COAD prognosis prediction.

**Methods:**

To uncover the roles of CSC in COAD tumorigenesis, chemoresistance, and prognosis, we retrieved COAD patients’ RNAseq data from TCGA (The Cancer Genome Atlas). We further performed analysis of differentially expressed genes (DEGs) and mRNA expression-based stemness index (mRNAsi) to identify stemness-related COAD biomarkers. We then evaluated the roles of mRNAsi in tumorigenesis, clinical-stage, overall survival (OS), and chemoresistance. Afterward, we used identified prognostic stemness-related genes (PSRGs) to construct a prediction model. After constructing the prediction model, we used elastic Net regression and area under the curve (AUC) to explore the prediction value of PSRGs based on risk scores and the receiver operator characteristic (ROC) curve. To elucidate the underlying interconnected systems, we examined relationships between the levels of TFs, PSRGs, and 50 cancer hallmarks by a Pearson correlation analysis.

**Results:**

Twelve thousand one hundred eight DEGs were identified by comparing 456 primary COADs and 41 normal solid tissue samples. Furthermore, we identified 4351 clinical stage-related DEGs, 16,516 stemness-associated DEGs, and 54 chemoresistance-related DEGs from cancer stages: mRNAsi, and COAD chemoresistance. Compared to normal tissue samples, mRNAsi in COAD patients were marked on an elevation and involved in prognosis (*p =* 0.027), stemness-related DEGs based on chemoresistance (OR = 3.28, *p* ≤ 0.001) and AJCC clinical stage relating (OR = 4.02, *p* ≤ 0.001) to COAD patients. The prediction model of prognosis were constructed using the 6 PSRGs with high accuracy (AUC: 0.659). The model identified universal correlation between NRIP2 and FDFT1 (key PRSGs), and some cancer related transcription factors (TFs) and trademarks of cancer gene were in the regulatory network.

**Conclusion:**

We found that mRNAsi is a reliable predictive biomarker of tumorigenesis and COAD prognosis. Our established prediction model of COAD chemoresistance, which includes the six PSRGs, is effective, as the model provides promising therapeutic targets in the COAD.

**Supplementary Information:**

The online version contains supplementary material available at 10.1186/s12863-022-01063-9.

## Introduction

Colorectal cancer (CRC) is highly prevalent worldwide [1], and its incidence and mortality rate continue to rise [2]. Despite the advances in CRC diagnosis and treatment, prognosis of the disease is still underdeveloped due to recurrence, metastasis, and drug resistance [3]. CRC’s 5-year relative survival rate in non-metastatic patients is 90%, while in metastatic CRC (mCRC) patients [4] it is 14%. Therefore, more studies in this field should explore the mechanisms of tumorigenesis and chemotherapy resistance in CRC [5], and they should also provide scientific basis for developing a more effective prognosis factors and therapeutic targets.

Because tumors are comprised of tumor cells, cancer stem cells (CSCs) and microenvironment cells [6], CSCs are believed to contribute to the development and maintenance of tumors. CSCs not only contribute to the development of tumors, they also facilitate resistance to tumor treatments [7]. Colon cancer stem cells (CCSCs) facilitates colon cancer recurrence, metastasis, and resistance [8]. However, the properties and biomarkers of CCSCs have not been well understood.

We first explored DNA methylation-based stemness index (mDNAsi) and mRNA stemness index (mRNAsi) based on oncogenic dedifferentiation. Our results show that mRNAsi mirrors stemness gene expression while mRNAsi indicates stemness epigenetic characteristics [9, 10]. These indexes greatly influenced the CSCs activity, loss of specialization, and pathologic grading [11]. However, mRNAsi’s role in CRC and chemotherapy resistance are still unclear.

In this current study, we retrieved published RNAseq data and patients’ clinical information from TCGA. We also identified differential expressed genes (DEGS) based on chemotherapy resistance, overall survival (OS) of patients, mRNAsi, and tumorigenesis. From these data, we constructed a prognostic prediction model using prognostic stemness-related genes (PSRGs) in our study to determine therapeutic targets and prognostic biomarkers of COAD. The identified PSRGs regulatory networks and downstream signaling pathways could provide clinicians a basis for preventing COAD occurrence and metastasis.

## Methods

### Data sources and extraction

RNAseq data on 456 primary COAD issues and 41 adjacent normal samples, including raw counts, (FPKM values), were retrieved from TCGA using the bio links package (v2.18.0) on R (v4.0.2). We also obtained Chemoresistance, diagnosis, demographics, tumor information, and endpoint data. All methods were performed in processing the database were in accordance with the TCGA relevant guidelines and regulations.

### mRNAsi estimation

We used one-class logistic regression machine learning (OCLR) algorithm, which applies standardized gene expression profiles to each sample, to determine mRNAsi. The mRNAsi, reported by Malta, T. M, is an activity assessing index between 0 and 1. In this study, we presented the results of COAD in Table S1 (Stemness Indices Derived for All PanCancer 33 TCGA Cohort.xlsx).

### Functional enrichment analysis and determination of differentially expressed genes

Four groups of DEGs were analyzed using DESeq2 using false discovery rate (FDR) ≤ 0.05 and |log2 fold change (FC)| > 1.0. The 4 groups include low mRNAsi COAD vs high mRNAsi COADs (divided by median mRNAsi), stage I/II COAD vs stage III/IV COAD, chemosensitive COAD vs chemoresistant COAD, and primary COAD vs normal adjacent tissue. The analyses we used to investigate the communication of signals in oncogenesis, cancer progression, and chemoresistance [12] were GO, KEGG, and GSEA. We used the GO term analysis to reveal molecular functions (MFs), cellular components (CCs), and biological processes (BPs) of enriched differential expression genes. We used KEGG pathway analysis to explore enriched pathways [13–15], also collecting fifty cancer gene set hallmarks from the molecular signatures database (MSigDB v7.2.1).

### PRSGs identification

Six DEGs were subjected to univariate Cox regression analysis (survival, v3.1.12), so we have identified PRSGs using *p* ≤ 0.05 as the cutoff. They were then subjected to multivariate Cox analysis using elastic net regression analysis to avoid overfitting. This was then followed by a ten-fold cross-validation (glmnet v4.1). We then explored five-year OS using the AUC of ROC curve, discrimination, and goodness of fit (GOF). The multivariate model was determined using a deviance-residual plot.

### Independent prognosis analysis and determination of prognostic index (PI)

PI was calculated using the formula:

$${PI}_m=\sum_{i=1}^n{\beta}_i{\mathrm{PRSG}}_i$$

“ *m *” represents one of the COAD patients, “ *β* ” indicates the coefficient of each PRSG, and “ *n* ” represents the number of prognostic PRSGs in the multivariate model. COAD patients were grouped into either a high-risk or low-risk group based on median PI. Kaplan-Meier survival analysis overestimates the independent prognosis value of the PI in COAD. Therefore, AJCC TNM stage gender and a year of diagnosis were adjusted for univariate Cox regression analysis and multivariate Cox regression.

### Development of a prognostic nomogram

A prognostic nomogram for this prediction of 3- and 5-year COAD survival was constructed using Cox models, including PI, and standardized using calibration plots (rms, v6.2.0).

### Analysis of signaling pathways and transcription factors associated with PRSGs

We first obtained official gene signs of 318 cancer-related TFs and 50 cancer gene set hallmarks from the Cistrome database (http://cistrome.org/) and molecular signatures database (MSigDB, v7.2.1, https://www.gsea-msigdb.org/gsea/msigdb/index.jsp), respectively [16]. The absolute activity of cancer gene markers was quantified using GSVA gene set variation analysis [17]. Next, we performed co-expression analysis on the absolute quantification of 50 hallmarks of cancer, numbers of TFs, and numbers of PRSGs. Then, we also performed a Co-expression network analysis among the PRSGs, TFs, and hallmarks of cancer using a *P*-value < 0.05 and a correlation coefficient > 0.30.

### Validating the prognostic stemness-related genes protein expression levels and prognostic significance

We examined the protein expression levels of six PRSGs using the Human Protein Atlas (HPA) online data [18]. We also tested the ability of the stemness-related gene constructed model to predict prognosis by using two chip data sets: GSE39582 dataset (https://www.ncbi.nlm.nih.gov/geo/query/acc.cgi?acc=GSE39582) and GSE17538 dataset (https://www.ncbi.nlm.nih.gov/geo/query/acc.cgi?acc=GSE17538).

### Statistical analysis

Variables, that were not continuously distributed, were expressed as percentages. Normally, we would use distributed continuous variables as mean ± SD or median (range), but we compared the differences between normally distributed continuous variables using a student t-test. We used Mann-Whitney U-test and Kruskal-Wallis H-test for not normally distributed data. The odds ratio (OR) and 95% confidence interval (95% CI) were also determined using Fisher’s exact test. We used *P* ≤ 0.05 (two-sided) to represent statistical significance, and performed statistical analysis using the R software (https://www.gsea-msigdb.org/gsea/msigdb/index.jsp).

## Results

### Identification of differentially expressed genes and functional enrichment analysis

**Fig. 1:**
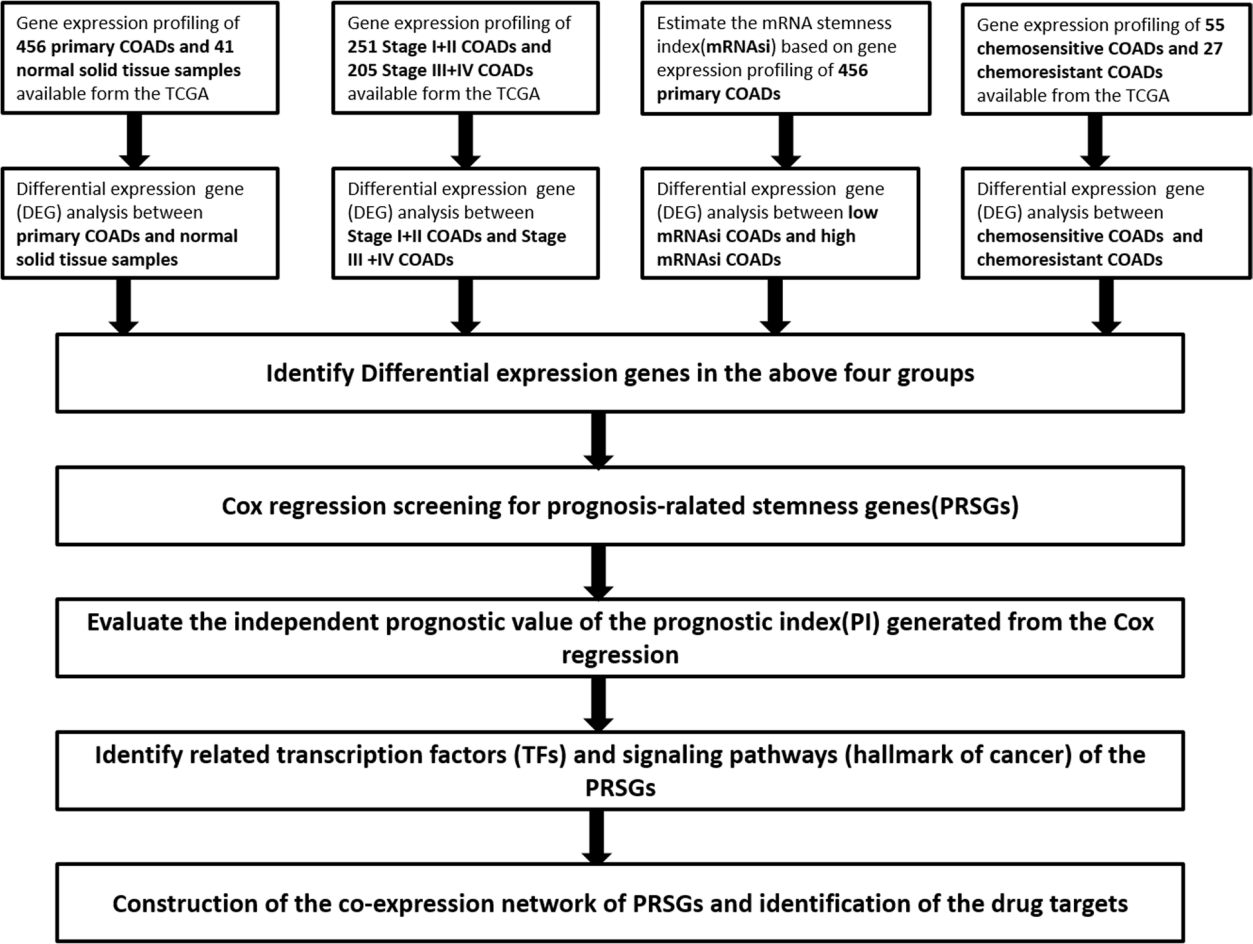
Experimental design for the study

**Fig. 2:**
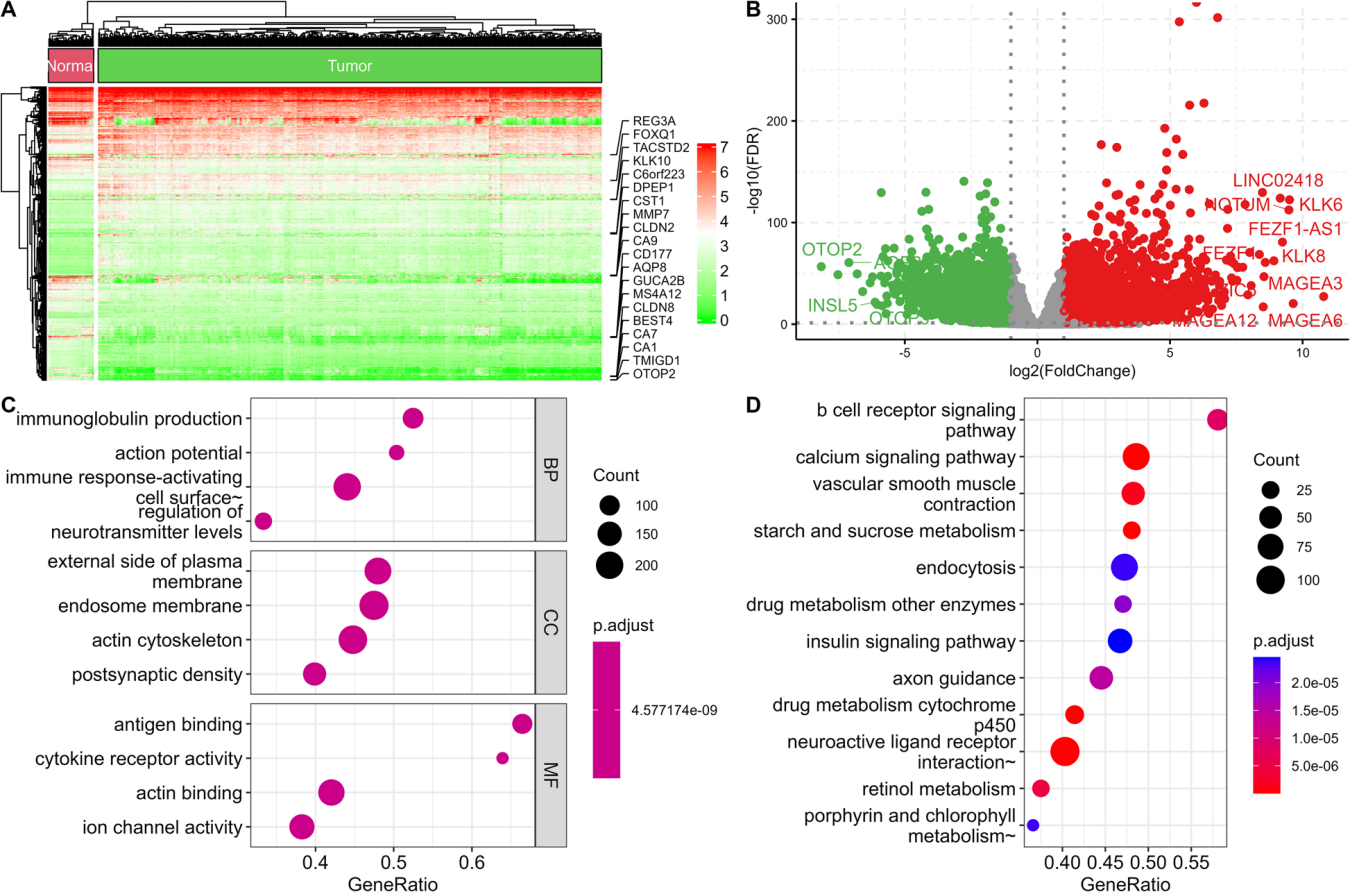
Functional enrichment analysis and differential gene expression analysis between primary COADs and adjacent normal samples. Differentially expressed genes are presented as a heatmap (**A**) and a volcano plot (**B**). GO (**C**) and KEGG (**D**) terms are also associated with differentially expressed genes

The study design, presented in Fig. 1, included only samples with clinicopathological information: clinical-stage, mRNAsi level, and chemotherapy sensitivity. We identified a total of 12,108 DEGs (4605 downregulated and 7503 upregulated) by comparison between 456 primary COADs and 41 normal tissues. We used heatmaps and volcano plots (Fig. 2A-B) to visualize this comparison. Then the 12,108 DEGs’ distinctive properties were analyzed by GO and KEGG analysis. Also, the significantly enriched cellular components (CCs), biological processes (BPs), and molecular functions (MFs) terms included regulation of neurotransmitter levels, external side of the plasma membrane, and actin-binding, respectively (Fig. 2C). KEGG pathway analysis showed calcium signaling, drug metabolism cytochrome p450, neuroactive ligand-receptor interaction, and starch and sucrose metabolism were significantly enriched (Fig. 2D).

**Fig. 3:**
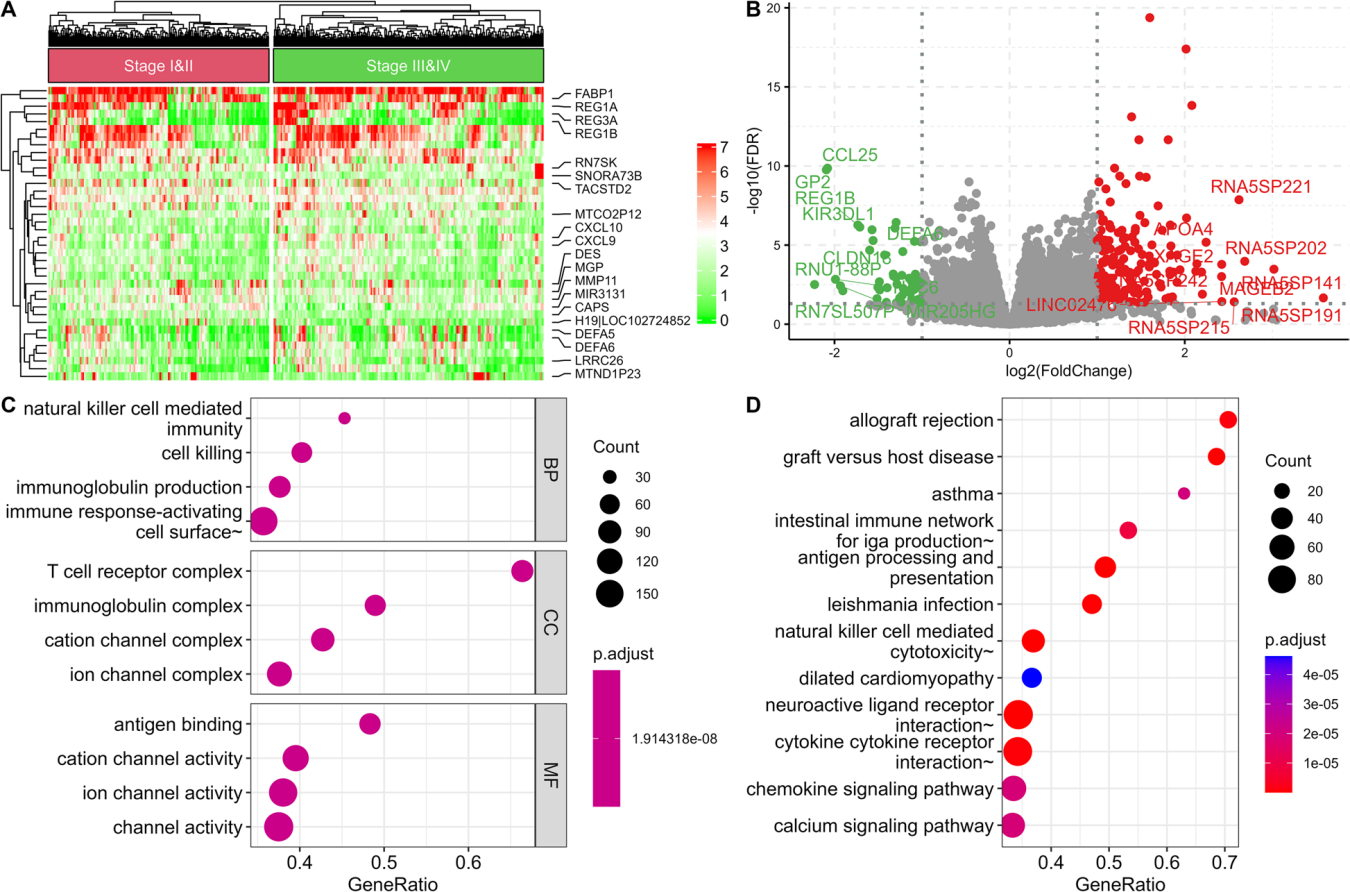
Functional enrichment analysis and differential gene expression analysis in stage I vs II COAD and stage III vs IV COAD. Differentially expressed genes are expressed as a heatmap (**A**) and a volcano plot (**B**). GO (**C**) and KEGG (**D**) terms are also associated with differentially expressed genes

Of the 456 primary COAD cases, 251 were in stage I/II, and 205 were in stage III/IV COADs. While comparing stages I/II and III/IV, we found that 4351 genes (1531 downregulated and 2820 upregulated) were differentially expressed (Fig. 3A-B). GO analysis revealed cell killing, immunoglobulin complex, and antigen-binding as significantly enriched terms (Fig. 3C). KEGG pathway analysis that showed chemokine signaling, natural killer cell-mediated cytotoxicity, antigen processing and presentation, and neuroactive ligand-receptor interaction were significantly enriched (Fig. 3D).

**Fig. 4:**
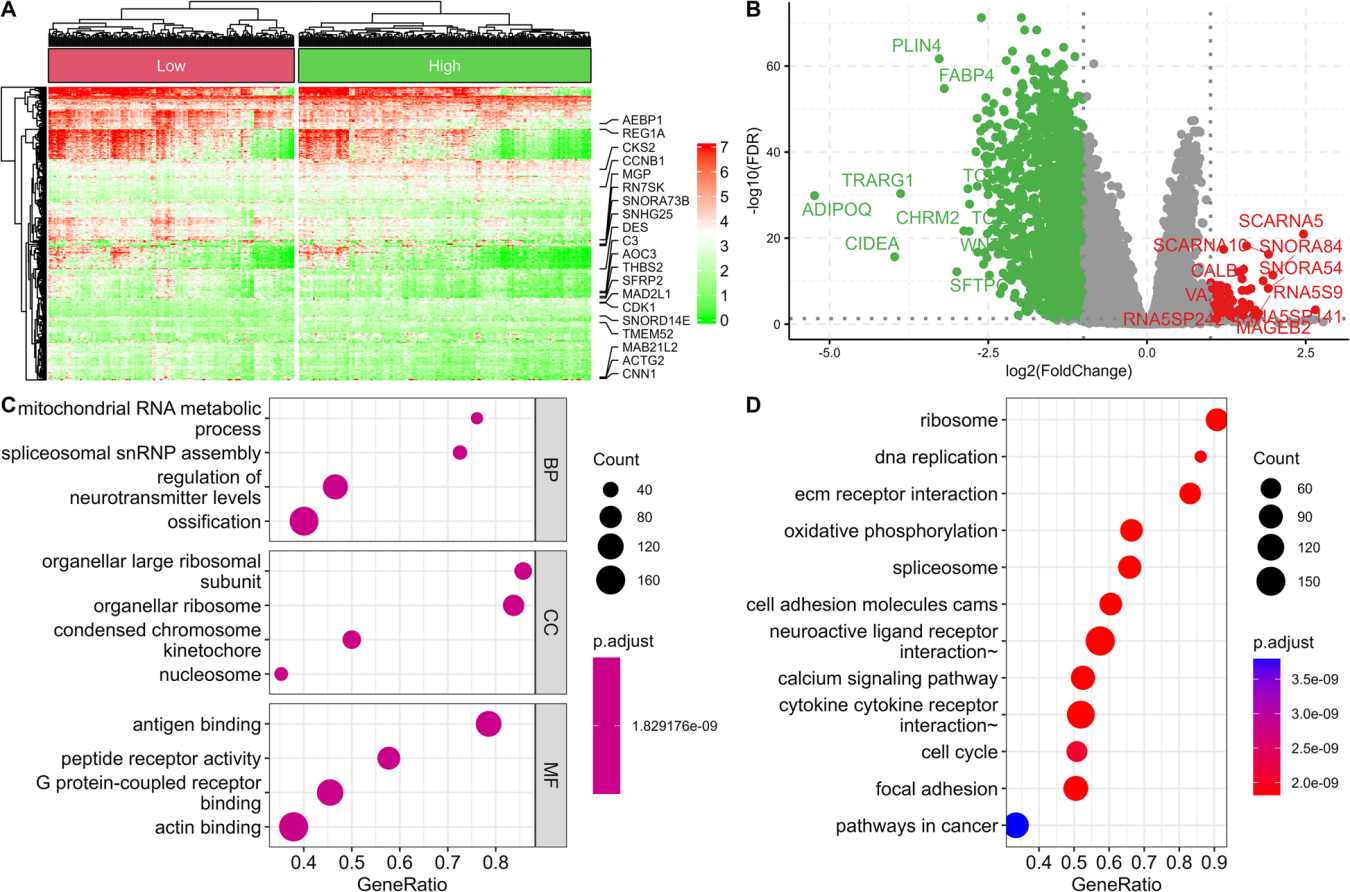
Functional enrichment analysis and differential gene expression analysis between low mRNAsi COAD and high mRNAsi COAD. Differentially expressed genes are presented as a heatmap (**A**) and a volcano plot (**B**). GO (**C**) and KEGG (**D**) terms are also associated with differentially expressed genes

We also identified a total of 16,516 stemness DEGs (9435 downregulated and 7081 upregulated) based on mRNAsi level (Fig. 4A, B). GO revealed spliceosomal snRNP assembly, organellar ribosome, and peptide receptor activity as significantly enriched terms for MFs, BPs, CCs, and BPs correlated with stemness (Fig. 4C). KEGG pathway analysis identified calcium signaling, cell adhesion molecules, and DNA replication as pathways involved in cancer stemness (Fig. 4D).

**Fig. 5:**
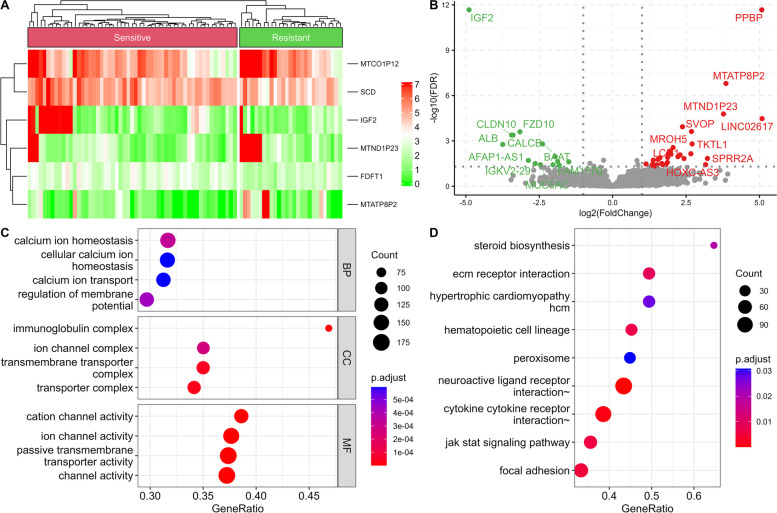
Functional enrichment analysis and differential gene expression analysis in chemosensitive and chemoresistant COADs. Differentially expressed genes are presented as a heatmap (**A**) and a volcano plot (**B**). GO (**C**) and KEGG (**D**) terms are associated with differentially expressed genes

Among the 456 primary COAD cases, there were 55 chemotherapies sensitive cases and 27 resistant cases. By comparing the two COAD cases, we further identified a total of 54 chemoresistance-related DEGs (17 downregulated and 37 upregulated) (Fig. 5A-B). GO revealed that the chemoresistance-associated DEGs can be enriched for calcium ion homeostasis, immunoglobulin complex, and ion channel activity (Fig. 5C). KEGG pathway analysis also revealed that the chemoresistance-specific DEGs can significantly enriched for JAK/STAT signaling pathway cytokine-cytokine receptor interaction, and neuroactive ligand-receptor interaction (Fig. 5D).

### Correlation of mRNAsi with clinical characteristics

**Fig. 6:**
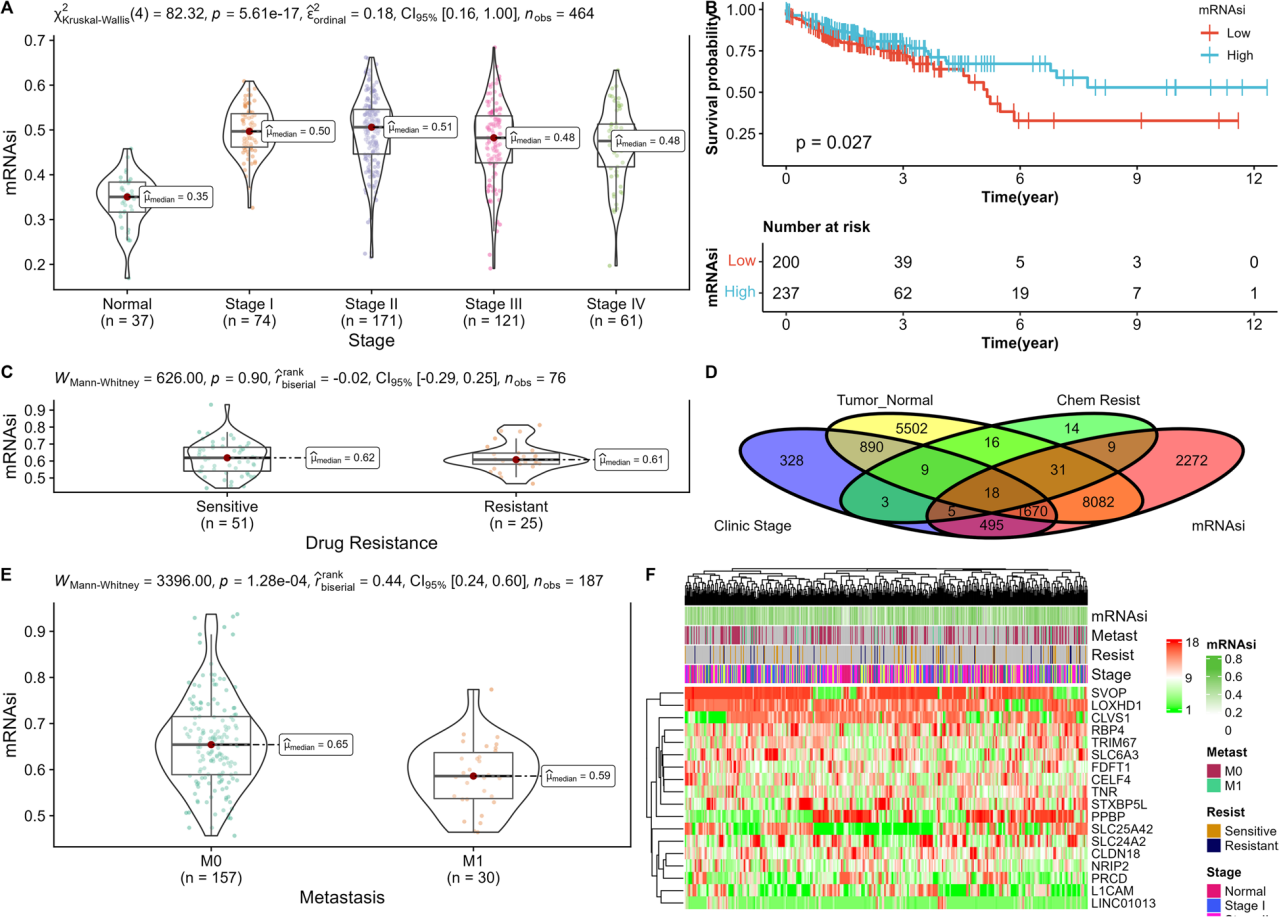
Clinical significance of mRNAsi and determination of stemness-related genes. **A** mRNAsi differences in normal vs tumor group at various clinical stages. **B** Kaplan-Meier survival analysis of COADs with mRNAsi groups. **C** mRNAsi differences in chemosensitive vs chemoresistant groups. **D** Tumorigenesis, stemness, clinical stage, and chemoresistance related differential gene expression are presented as a Venn plot. **E** mRNAsi differences among metastases. **F** Heatmap of mRNAsi with chemoresistance, metastasis, and tumor stages

Venn diagram showed that 18 DEGs were involved in all four signs of progresses including tumorigenesis, stemness, clinical-stage differentiation, and chemoresistance. Eight DEGs of the 18 DEGs were expressed increasingly in the tumor (Fig. 6D). In addition, compared to adjacent normal solid tissue, mRNAsi was increasingly regulated abnormally in primary COAD (*p ≤* 0.001, Fig. 6A) based on non-parametric (Mann-Whitney U-test or Kruskal-Wallis H-test) and Kaplan-Meier survival analysis. mRNAsi was also remarkably involved in the prognosis of COAD patients (*p* = 0.027, Fig. 6B), as stemness-related DEGs were correlated with chemoresistance (OR = 3.28, *p ≤* 0.001, Table 1) and AJCC clinical stage (OR = 4.02, *p ≤* 0.001, Table 2). Interestingly, mRNAsi had no difference between chemosensitive and chemoresistant COADs (*p* = 0.903, Fig. 6C), but mRNAsi had a relationship with low levels in metastasis cases(*p ≤* 0.001, Fig. 6E). Thus, we could visualize the levels of the 18 genes on a heatmap (Fig. 6F).

**Table 1:** Distribution of genes in stemness and chemoresistance-associated groups

	Chemoresistance-associated		OR(95%CI)	***P***-value
Stemness-associated	No	Yes		
**No**	40,063	23	1.0	
**Yes**	16,485	31	3.28 (1.85,5.88)	1.63e-05

**Table 2:** Distribution of genes in stemness and progress-associated groups

	Chemoresistance-associated		OR(95%CI)	***P***-value
Stemness-associated	No	Yes		
**No**	38,320	1766	1.0	
**Yes**	13,931	2585	4.03 (3.78,4.29)	< 2.2e-16

### Identification of PRSGs and analysis of independent prognosis

**Fig. 7:**
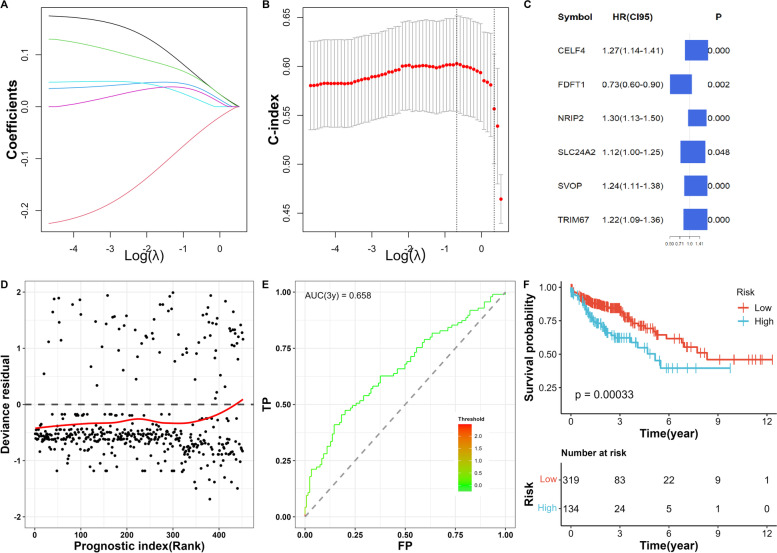
Multivariate Cox model based on prognostic stemness-related genes. **A** Elastic net regression analysis of stemness-related genes (**A**, **B**). **C** Multivariate model based on 6-prognostic stemness-related genes. **D** Residual plot of the multivariate model and ROC curve (**E**). **F** Kaplan-Meier analysis based on the risk score

As we combined the univariate Cox regression analysis, 18 DEGs, PRSGs, and genes with prognostic values (Fig. 7A, B), we identified and incorporated this information into the Elastic Net regression analysis (model parameters: α = 0.0417, β = 1.7022). Results showed that only 6 PRSGs (NRIP2, FDFT1, CELF4, SLC24A2, TRIM67, and SVOP) were essential for fitting models (Fig. 7C).

The prognosis index (PI) for each COAD patient was determined as described in the methods section. The residual plots and three-year OS ROC curves (AUC = 0.658) showed good discrimination and GOF of the multivariate Cox regression model (Fig. 7D, E). The prognostic potential of PI in COAD patients was determined using Kaplan-Meier survival analysis (Fig. 7F, *p ≤* 0.001).

### Prognostic nomogram construction

**Fig. 8:**
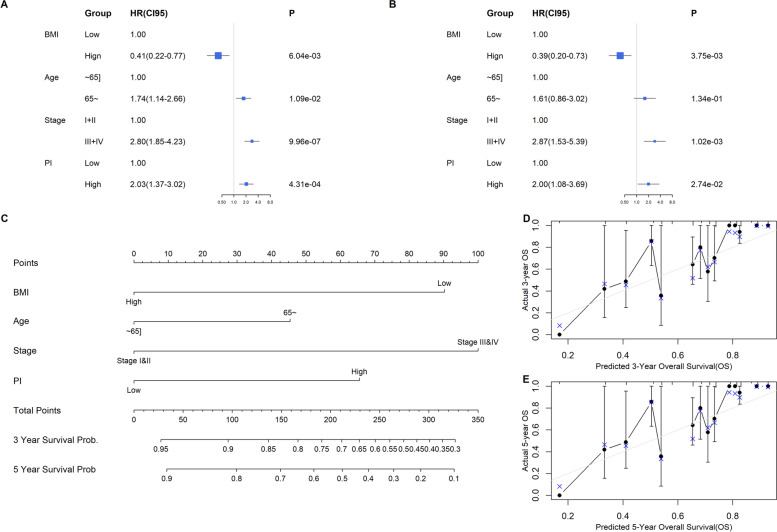
Prognostic independent analysis and development of nomogram for COAD. Univariate (**A**) and multivariate (**B**) Cox regression models adjusted by demographics and histologic grade. **C** A prognostic nomogram based on the multivariate Cox model. **D**, **E** The calibration curve indicated acceptable nomogram calibration

Univariate (HR =2.03, 95% CI: 1.37, 3.02, *p ≤* 0.001) and multivariate (HR = 2.00, 95% CI: (1.08, 3.69), *p* = 0.027) Cox analyses adjusted for patient demographics and AJCC clinical stage showed PI as an independent predictor of COAD prognosis (Fig. 8A and B).

We constructed a model, using the Cox model, for predicting OS by prognostic nomogram (Fig. 8C), and we also determined the acceptable calibration (Fig. 8D-E) of the prognostic nomogram by calibration curve analysis. Based on bioinformatics and clinical research requirements, we should adjust gene expression levels by demographics by constructing a multivariate regression model; age and BMI are analyzed as categorical variables in some studies. Notably, gender was inappropriate for nomogram construction since it was not significant based on multivariate Cox analysis. Other important clinical-pathological characteristics like primary diagnosis, grade, clinical T/N/M classification, and histology subtype were excluded from the multivariate regression model to preserve more modeling samples. Finally, we used BMI, age, clinic stage, and PI to plot the nomogram. We included PI because it was an independent predictor of COAD prognosis as determined by multivariate regression analysis.

### Identification of PRSGs co-expressed TFs and related signaling pathways

**Fig. 9:**
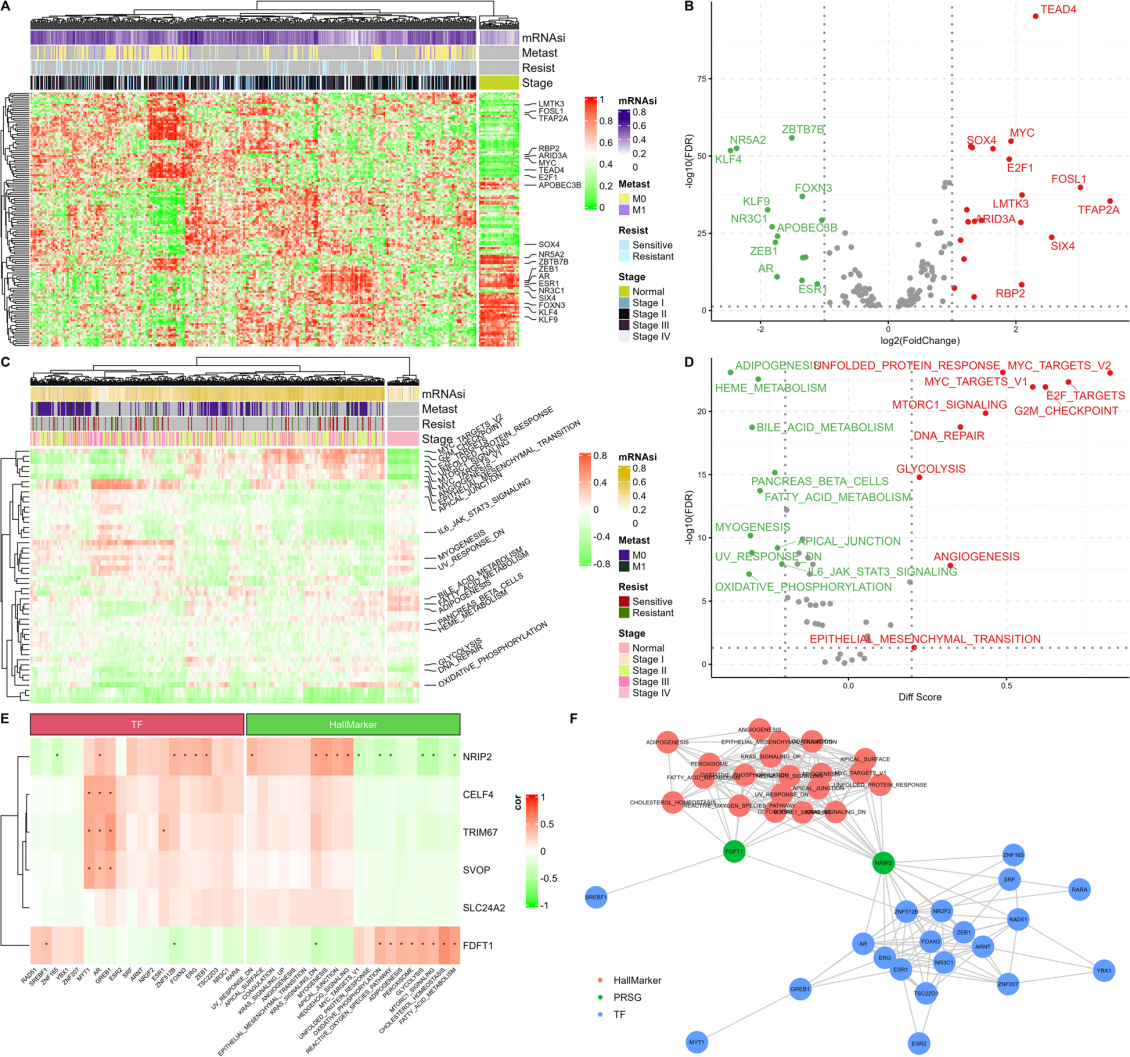
PRSGs co-expressed TFs and hallmarks of cancer gene sets. Heatmap (**A**) and volcano plot (**B**) of differentially expressed TFs in cancer vs normal, adjacent samples. Heatmap (**C**) and volcano plot (**D**) of differentially expressed hallmarks of cancer gene sets in cancer vs normal solid tissue samples. A comparison of co-expression analysis (**E**) and co-expression interaction pairs (**F**) between PRSGs, TFs and hallmarks of cancer

Our findings showed 133 cancer-associated TFs (Fig. 9B) in primary COADs and adjacent normal tissues. The TFs, MYC, SOX4, E2F1, and TEAD4 were upregulated, while KLF4, NR5A2, and AR were downregulated in COAD. Interactions between the TFs and the 50 cancer gene sets hallmarks with mRNAsi, chemoresistance, tumor stage, and metastasis were presented as heatmaps (Fig. 9A and C). Expression makers of primary COADs compared to adjacent normal tissues were visualized in volcano plots (Fig. 9D). The volcano plots revealed MYC, G2M checkpoint, and DNA repair angiogenesis signaling pathways to be overexpressed. We performed Co-expression analysis to explore relationships among hallmarks of cancer, TFs, and PRSGs. We also constructed a co-expression network based on interaction pairs between hallmarks or TFs and PRSGs with *p ≤* 0.05 and |correlation coefficients | > 0.30, respectively. By using a total of 20 TFs that met the criteria, we can establish a regulatory network with 6 PRSGs (Fig. 9E). Figure 9C shows the abundance of KRAS signaling, adipogenesis, unfolded protein response, cholesterol homeostasis oxidative phosphorylation, reactive oxygen species pathway, mTORC1 signaling, and peroxisome. Our findings showed that NRIP2 and FDFT1 were hub genes, and they were possibly stemness-related targets in the chemoresistance of COAD (Fig. 9F).

### Validating the protein expression levels and the predictive value of prognostic stemness-related genes in GSE cohorts

**Fig. 10:**
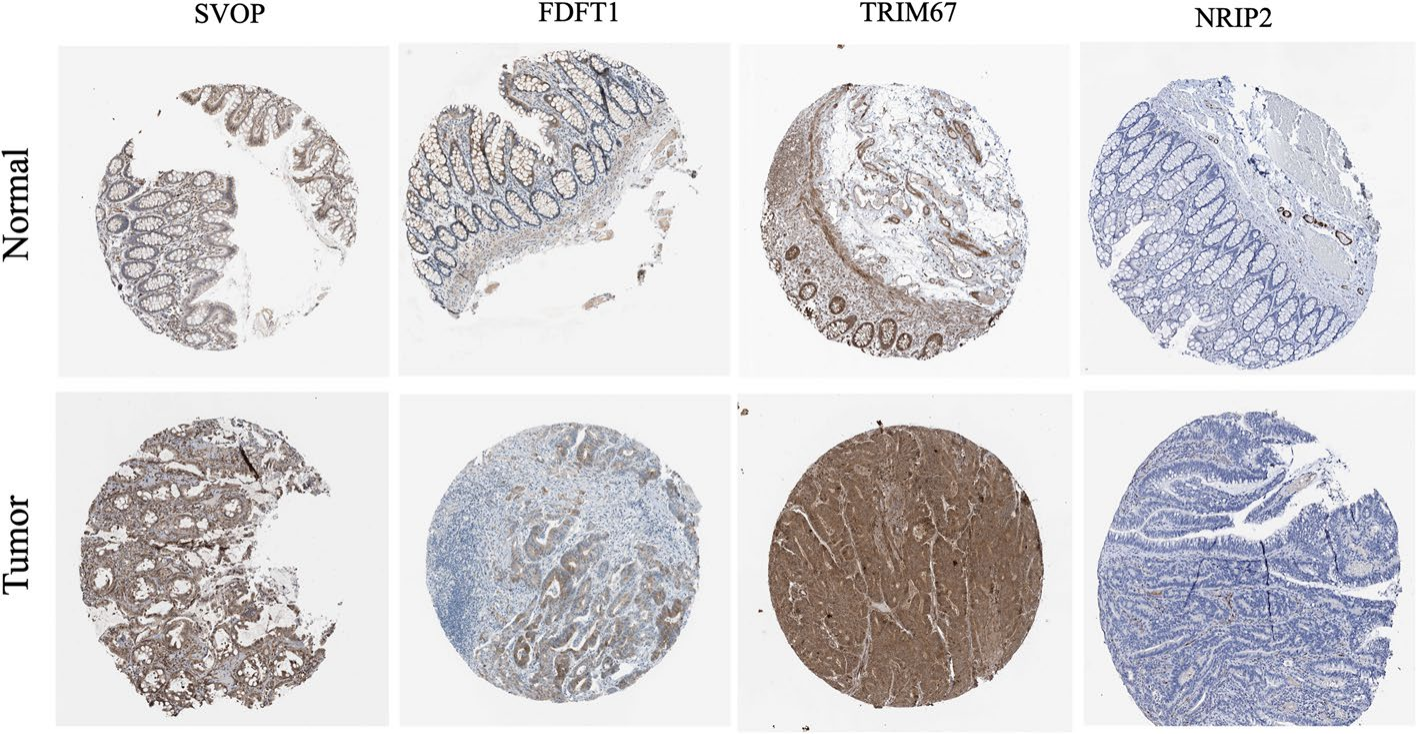
The protein levels of PRSGs in COAD tissues and colon normal tissue using the Human Protein Atlas (HPA) database

**Fig. 11:**
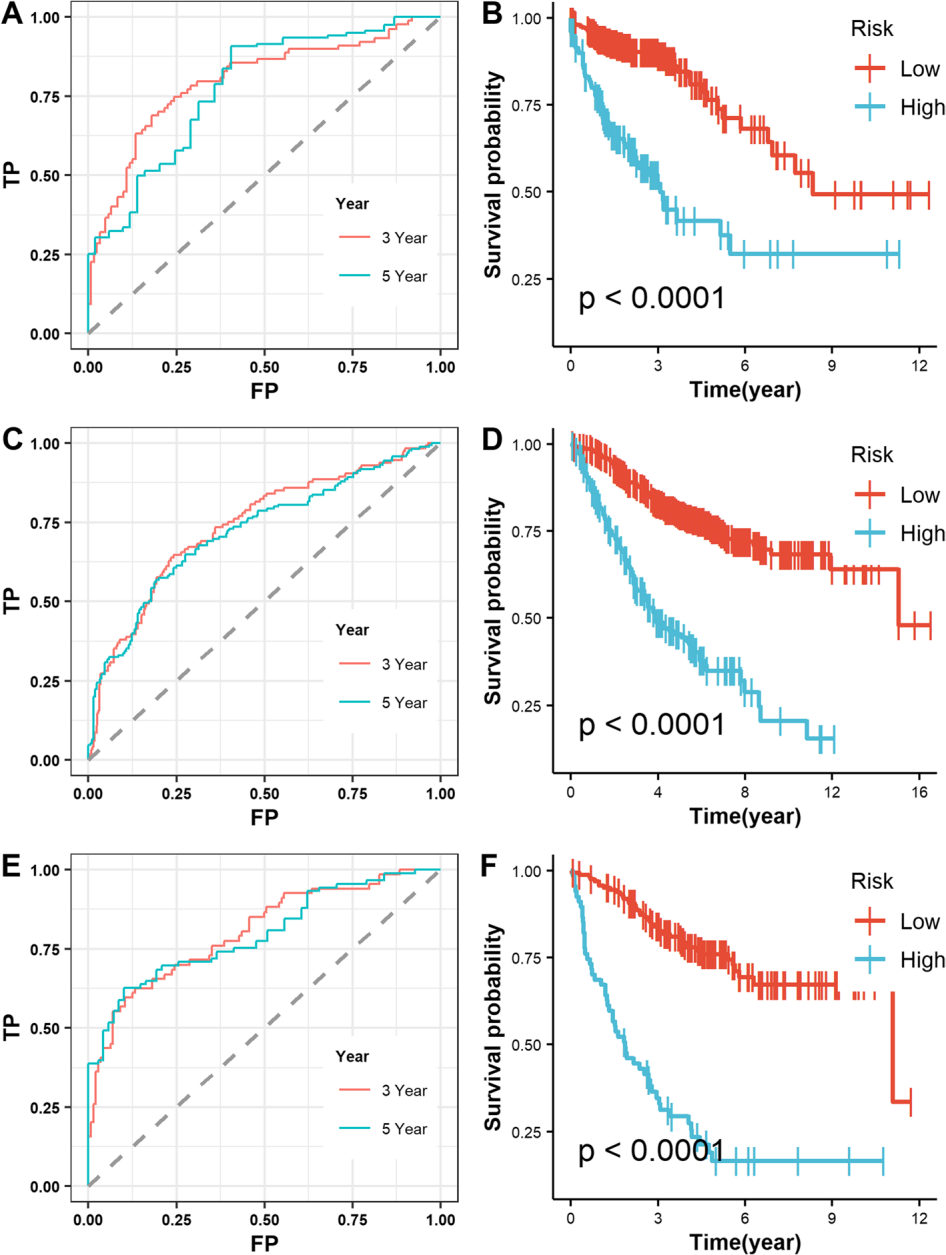
**A** ROC curve of the six-gene signature plus age, gender, and stage in TCGA discovery cohort; (**B**) KM curve of the prognostic index (PI) in TCGA discovery cohort; (**C**) ROC curve of GSE39582 external validation cohort; (**D**) KM curve of PI in GSE39582 external validation cohort; (**E**) ROC curve of GSE17538 external validation cohort; (**F**) KM curve of PI in GSE17538 external validation cohort

The Human Protein Atlas database analyzed the expression of six key PRSGs, the analysis contains immunohistochemical results for four PRSGs (not including CELF4 and SLC24A2) in COAD tissues and colon normal tissues (Fig. 10). We discovered that SVOP, FDFT1, and TRIM67 were significantly higher in COAD tissues than in colon normal tissue (Fig. 10), and also, NRIP2 was barely expressed in COAD tissues and colon normal tissue in the HPA dataset, while NRIP2 was reported to be up-regulated in CCICs from both cell lines and primary colorectal cancer tissues [19]. To further assess the clinical significance of six key PRSGs, we used the GSE39582 and GSE17538 external validation cohorts to confirm the prediction ability of the prognosis model constructed by stemness-related genes. According to further assessment, we found that high expression of PRSGs was significantly (*p* < 0.001) associated with poor prognosis (Fig. 11).

## Discussion

Colorectal cancer is highly prevalent worldwide and can cause high mortality rate in end-stage patients due to metastasis, recurrence, and drug resistance [20, 21]. CSCs, the main driver of poor COAD prognosis, are often activated from a dormant state by radiotherapy or chemotherapy, and it can promote tumor invasion, metastases, and enhancing chemotherapy resistance [22]. In this experiment, we uncovered the relationships between clinical stage, mRNAsi and COAD chemoresistance. Therefore, we identified increasing mRNAsi expression in primary COAD and its relationship with prognosis. Stemness-related DEGs also correlates with chemoresistance and AJCC clinical stage. Additionally, a highly accurate model, including 6 PSRGs, was successfully constructed to predict COAD prognosis, and also NRIP2 and FDFT1 were hub genes, which means they are possibly stemness-related targets in the chemoresistance of COAD.

CSCs are potential drivers of CRC recurrence after treatment [23] as remaining tumor stem cells can maintain their own stem cell characteristics and also CSCs can promote further tumor growth. Thus, CSCs initiate the tumor recurrence and metastasis. Single-cell transcriptome integrated with telomere length detection technology has also revealed that CCSCs are in a resting state and have relatively short telomere length [24]. But in some conditions, CCSCs transform into fast-growing tumor cells with extended telomeres. Thus, these resting tumor stem cells may be major drivers of tumor recurrence and drug resistance. KRT18, CLDN4, CXADR, and SLC12A2 are the potential cellular functions of new prognosis-valuable genes.

Here, CSCs features were identified using mRNAsi, a significant association between them and oncogenesis, prognosis, AJCC clinical stage, and drug resistance in COAD. The roles of CSCs in COAD chemoresistance may offer more ways to optimize drug-resistance monitoring systems and curing COAD. Six key PRSGs (NRIP2, FDFT1, CELF4, SLC24A2, TRIM67, and SVOP) were identified using a multivariate Cox model and associated analyses. Among the six key PRSGs, NRIP2 and FDFT1 correlate with many cancer-associated TFs and hallmarks of cancer gene sets in the regulatory network. FDFT1, a membrane-associated enzyme, is also crucial for cancer development [25], particularly in metabolic reprogramming. FDFT1 may be a promising predictor of CRC prognosis and might offer vital implications for targeted therapy or immunotherapy [26]. Right now, NRIP2 is upregulated in colorectal cancer initiating cells (CCIC) and mediates CCIC self-renewal via Wnt signaling [27]. CELF4, one of the CELF protein family, which belongs to a group of splicing regulators that controls developmentally regulated and tissue-specific splicing events [28]. SLC24A2, a potassium-dependent sodium-calcium exchanger, were observed in pancreatic ductal adenocarcinoma and were associated with esophageal squamous cell carcinoma prognosis [29]. TRIM67 was involved in neuroprotective effects and tumor processes, has been reported as a potential target to inhibit CRC metastasis [30]. SVOP, a transporter-like protein, and involved in neuron formation, maturation, and neuronal function. SVOP aberrant methylated played an important role in regulation of nervous system, and was associated with progression of glioblastoma [31].

A model with relatively high accuracy (AUC: 0.659) was built for predicting COAD overall survival based on the 6 PSRGs model. Many previous studies aimed to identify the prognostic biomarkers and the prediction model in COAD patients (CXC Chemokine-Based Prediction Model [32], non-invasive imaging prediction model for liver metastasis) which facilitates decision-making in COAD care. Various prediction models have been used to predict COAD prognosis, but there prediction models did not report CSC-related signatures and PSRGs. Thus, our findings offer vital and novel insights into COAD prognosis.

We know that, comparably to normal stem cells, CSCs exhibit characteristics that could be associated with the expression of similar TFs [33]. Hence, we find that CSCs upregulated cells were MYC, SOX4, E2F1, and TEAD4 were upregulated while downregulated cells were KLF4, NR5A2, and AR in COAD. MYC is a potent oncogene with numerous biological functions that contribute to tumorigenesis [34]. MYC can promote bone marrow stem cell dysfunction [35] and leukemia stem cell self-renewal [36]. SOX4, a primary transcription factor, regulates stemness, differentiation, and progenitor development. SOX4 is frequently mutated and upregulated in more than 20 cancers [37]. E2F1 is a regulator of CML stem/progenitor cell proliferation. TEAD4 is one of the important member of the TEAD family, and was reported to be a innovate prognostic marker in many cancer which includes gastric cancer, breast cancer, colorectal cancer, melanoma [38]. KLF4 can reprogram of differentiated cells into pluripotent embryonic stem cells, and combats tumor growth and chemoresistance in hepatocellular carcinoma [39]. NR5A2 can bind the same DNA motif and plays crucial role in gonadal development and function and was associated with favorable prognosis in patients with glioblastoma and neuroblastoma tumors [40]. AR is a nuclear receptor that regulates gene expression programs required for prostate development and male phenotype maintenance [41]. We found that overexpressed TFs needs to be enriched for KRAS signaling, oxidative phosphorylation, reactive oxygen species, and mTORC1 signaling. Oxidative phosphorylation and mTORC1 signaling also are enriched in cancer stem cells. Therefore, we implicated the identified TFs and signaling pathways in COAD chemotherapy resistance.

## Conclusion

We established a prediction model using CSC-related genes and mRNAsi effectively and accurately predicts colon cancer prognosis. We also investigated the potential interconnection between cancer gene sets and key PSRGs to reveal their modulation in COAD chemotherapy resistance. However, the underlying mechanisms of the six PRSGs needs further experimental validation.

## Supplementary Information

**Additional file 1: Supplementary Table 1.** Stemness Indices Derived for All PanCancer 33 TCGA Cohort.

**Additional file 2: Supplementary Table 2.** The differentially expressed genes of tumor, stage, mRNAsi and chemoresistance.

## Data Availability

TCGA publicly available datasets were used in the current study. TCGA program website (https://portal.gdc.cancer.gov/).

## References

[1] Siegel RL, Miller KD, Jemal A (2020). Cancer statistics, 2020. CA Cancer J Clin.

[2] Kehm RD, Lima SM, Swett K (2021). Age-specific trends in colorectal cancer incidence for women and men, 1935-2017. Gastroenterology.

[3] Breuer E, Hebeisen M, Schneider M (2021). Site of recurrence and survival after surgery for colorectal peritoneal metastasis. J Natl Cancer Inst.

[4] Li J, Yuan Y, Yang F (2019). Expert consensus on multidisciplinary therapy of colorectal cancer with lung metastases (2019 edition). J Hematol Oncol.

[5] Di Franco S, Todaro M, Dieli F, Stassi G (2013). Colorectal cancer defeating? Challenge accepted!. Mol Asp Med.

[6] Kreso A, Dick JE (2014). Evolution of the cancer stem cell model. Cell Stem Cell.

[7] Dalerba P, Clarke MF (2007). Cancer stem cells and tumor metastasis: first steps into uncharted territory. Cell Stem Cell.

[8] Fumagalli A, Oost KC, Kester L (2020). Plasticity of Lgr5-negative Cancer cells drives metastasis in colorectal Cancer. Cell Stem Cell.

[9] Malta TM, Sokolov A, Gentles AJ (2018). Machine learning identifies Stemness features associated with oncogenic dedifferentiation. Cell.

[10] Rooks MG, Garrett WS (2017). One-class detection of cell states in tumor subtypes. Physiol Behav.

[11] Garvalov BK, Acker T (2011). Cancer stem cells: a new framework for the design of tumor therapies. J Mol Med.

[12] Shi J, Walker M (2008). Gene set enrichment analysis (GSEA) for interpreting gene expression profiles. Curr Bioinforma.

[13] Kanehisa M, Goto S (2000). KEGG: Kyoto encyclopedia of genes and genomes. Nucleic Acids Res.

[14] Kanehisa M (2019). Toward understanding the origin and evolution of cellular organisms. Protein Sci.

[15] Kanehisa M, Furumichi M, Sato Y, Ishiguro-Watanabe M, Tanabe M (2021). KEGG: integrating viruses and cellular organisms. Nucleic Acids Res.

[16] Liberzon A, Birger C, Thorvaldsdottir H (2016). The molecular signatures database (MSigDB) hallmark gene set. collection. Cell Syst.

[17] Hänzelmann S, Castelo R, Guinney J (2013). GSVA: gene set variation analysis for microarray and RNA-Seq data. BMC Bioinformatics.

[18] Li Y, Eresen A, Shang G (2019). Establishment of a new non-invasive imaging prediction model for liver metastasis in colon cancer. Am J Cancer Res.

[19] Ma YS, Wu ZJ, Zhang HW (2019). Dual regulatory mechanisms of expression and mutation involving metabolism-related genes FDFT1 and UQCR5 during CLM. Mol Ther Oncolytics.

[20] Longley DB, Harkin DP, Johnston PG (2003). 5-fluorouracil: mechanisms of action and clinical strategies. Nat Rev Cancer.

[21] Preet R, Mohapatra P, Satapathy SR, Kundu CN (2013). 1,3-bis (2-chloroethyl)-1-nitrosourea enhances the inhibitory effect of resveratrol on 5-fluorouracil sensitive/resistant colon cancer cells. World J Gastroenterol.

[22] Touil Y, Lgoudjil W, Corvaisier M (2014). Colon cancer cells escape 5FU chemotherapy-induced cell death by entering stemness and quiescence associated with the c-yes/YAP axis. Clin Cancer Res.

[23] Mathonnet M, Perraud A, Christou N (2014). Hallmarks in colorectal cancer: angiogenesis and cancer stem-like cells. World J Gastroenterol.

[24] Wang H, Gong P, Chen T (2021). Colorectal Cancer stem cell states uncovered by simultaneous single-cell analysis of transcriptome and telomeres. Adv Sci.

[25] Ha NT, Lee CH (2020). Roles of farnesyl-diphosphate farnesyltransferase 1 in tumour and tumour microenvironments. Cells.

[26] Weng ML, Chen WK, Chen XY (2020). Fasting inhibits aerobic glycolysis and proliferation in colorectal cancer via the Fdft1-mediated AKT/mTOR/HIF1α pathway suppression. Nat Commun.

[27] Wen Z, Pan T, Yang S (2017). Up-regulated NRIP2 in colorectal cancer initiating cells modulates the Wnt pathway by targeting RORβ. Mol Cancer.

[28] Wang X, Sun CL (2016). CELF4 variant and anthracycline-related cardiomyopathy: a children’s oncology group genome-wide association study. J Clin Oncol.

[29] Zhang D, Qian C, Wei H, Qian X (2020). Identification of the prognostic value of tumor microenvironment-related genes in esophageal squamous cell carcinoma. Front Mol Biosci.

[30] Liu Y, Wang G, Jiang X (2020). TRIM67 inhibits tumor proliferation and metastasis by mediating MAPK11 in colorectal Cancer. J Cancer.

[31] Zhao J, Wang L, Kong D, Hu G, Wei B (2020). Construction of novel DNA methylation-based prognostic model to predict survival in glioblastoma. J Comput Biol.

[32] Liu K, Lai M, Wang S (2020). Construction of a CXC chemokine-based prediction model for the prognosis of Colon Cancer. Biomed Res Int.

[33] Pádua D, Figueira P, Ribeiro I, Almeida R, Mesquita P (2020). The relevance of transcription factors in gastric and colorectal Cancer stem cells identification and eradication. Front Cell Dev Biol.

[34] Lourenco C, Resetca D, Redel C (2021). MYC protein interactors in gene transcription and cancer. Nat Rev Cancer.

[35] Rodríguez A, Zhang K, Farkkila A (2021). MYC promotes bone marrow stem cell dysfunction in Fanconi Anemia. Cell Stem Cell.

[36] Zhang L, Li J, Xu H (2020). Myc-Miz1 signaling promotes self-renewal of leukemia stem cells by repressing Cebpα and Cebpδ. Blood.

[37] Moreno CS (2020). SOX4: the unappreciated oncogene. Semin Cancer Biol.

[38] Chen M, Huang B, Zhu L (2020). Structural and functional overview of TEAD4 in cancer biology. Onco Targets Ther.

[39] Jia X, Li L, Wang F (2022). DUB3/KLF4 combats tumor growth and chemoresistance in hepatocellular carcinoma. Cell Death Discov.

[40] Gkikas D, Stellas D, Polissidis A (2021). Nuclear receptor NR5A2 negatively regulates cell proliferation and tumor growth in nervous system malignancies. Proc Natl Acad Sci U S A.

[41] Wasmuth EV, Broeck AV, LaClair JR (2022). Allosteric interactions prime androgen receptor dimerization and activation. Mol Cell.

